# Sensory Neuron-Specific Deletion of TRPA1 Results in Mechanical Cutaneous Sensory Deficits

**DOI:** 10.1523/ENEURO.0069-16.2017

**Published:** 2017-03-13

**Authors:** Katherine J. Zappia, Crystal L. O’Hara, Francie Moehring, Kelvin Y. Kwan, Cheryl L. Stucky

**Affiliations:** 1 Department of Cell Biology, Neurobiology and Anatomy, Medical College of Wisconsin, Milwaukee, WI 53226; 2 Department of Cell Biology and Neuroscience, Rutgers, The State University of New Jersey, Piscataway, NJ 08854

**Keywords:** advillin, cold, mechanical, sensory neuron, somatosensation, TRPA1

## Abstract

The nonselective cation channel transient receptor potential ankyrin 1 (TRPA1) is known to be a key contributor to both somatosensation and pain. Recent studies have implicated TRPA1 in additional physiologic functions and have also suggested that TRPA1 is expressed in nonneuronal tissues. Thus, it has become necessary to resolve the importance of TRPA1 expressed in primary sensory neurons, particularly since previous research has largely used global knock-out animals and chemical TRPA1 antagonists. We therefore sought to isolate the physiological relevance of TRPA1 specifically within sensory neurons. To accomplish this, we used *Advillin-Cre* mice, in which the promoter for Advillin is used to drive expression of Cre recombinase specifically within sensory neurons. These *Advillin-Cre* mice were crossed with *Trpa1*^fl/fl^ mice to generate sensory neuron-specific *Trpa1* knock-out mice. Here, we show that tissue-specific deletion of TRPA1 from sensory neurons produced strong deficits in behavioral sensitivity to mechanical stimulation, while sensitivity to cold and heat stimuli remained intact. The mechanical sensory deficit was incomplete compared to the mechanosensory impairment of TRPA1 global knock-out mice, in line with the incomplete (∼80%) elimination of TRPA1 from sensory neurons in the tissue-specific *Advillin-Cre* knock-out mice. Equivalent findings were observed in tissue-specific knock-out animals originating from two independently-generated *Advillin-Cre* lines. As such, our results show that sensory neuron TRPA1 is required for mechanical, but not cold, responsiveness in noninjured skin.

## Significance Statement

To date, most studies on the function of transient receptor potential ankyrin 1 (TRPA1) have used either global knock-out animals or antagonists, which do not allow for tissue specificity in their interpretation. Given that TRPA1 is expressed in multiple cell types, cell-type-specific targeting is necessary to elucidate the mechanisms through which TRPA1 facilitates mechanosensation and other physiologic functions. Our results show that deletion of TRPA1 from sensory neurons is sufficient to induce significant impairment in baseline mechanical sensitivity but does not impact behavioral cold aversion. Beyond the important clarification that the expression of TRPA1 specifically within sensory neurons is necessary for mechanosensation in mouse, sensory neuron-specific TRPA1 knock-out animals will be beneficial in future studies focused on isolating additional tissue-specific functions of TRPA1.

## Introduction

The ion channel transient receptor potential ankyrin 1 (TRPA1) is a polymodal, nonselective cation channel that is activated by chemical, thermal, and mechanical stimuli (Bandell et al., 2004; Vilceanu and Stucky, 2010; Brierley et al., 2011; Wang et al., 2011; Zayats et al., 2013). Key ion channels expressed within mechanically-activated sensory neurons include Piezo2 and TRPA1 (Story et al., 2003; Julius, 2013; Ranade et al., 2014). Much work has been done to elucidate the methods through which various mechanosensitive channels, including Piezo1 and Piezo2 in mammals and MscL in bacteria, sense mechanical force. Evidence suggests some ion channels are intrinsically sensitive to deformation of a plasma membrane or simplified lipid bilayer (Blount et al., 1996; Coste et al., 2010; Kung et al., 2010; Ranade et al., 2014; Cox et al., 2016). Secondly, several ion channels can be activated following indirect mechanical stress to the cell membrane and associated cytoskeleton (Nilius and Honoré, 2012; Brohawn et al., 2014). As such, TRPA1 may indirectly sense forces transmitted through the cytoskeleton to TRPA1’s many intracellular ankyrin repeats (Corey et al., 2004; Paulsen et al., 2015).

Soon after its initial identification in sensory neurons, TRPA1 became a central focus in the study of pain and mechanical hyperalgesia (Story et al., 2003; Bautista et al., 2006) and was recognized as integral to mechanical sensitization in a number of pain states, including acute and chronic inflammation, nerve injury, and diabetic neuropathy (Eid et al., 2008; Lennertz et al., 2012; Bautista et al., 2013; Garrison and Stucky, 2014). TRPA1 is similarly important for baseline mechanosensation (Kwan et al., 2006; Vilceanu and Stucky, 2010).

Beyond its role in mechanosensation, TRPA1 has been subject to debate regarding its role in cold sensation. Cumulative evidence shows that TRPA1 contributes to cold hypersensitivity during injury (Obata et al., 2005; del Camino et al., 2010; Zhao et al., 2012); however, its contribution to baseline thermal sensation is less clear, possibly due to functional similarly to other cold-sensing ion channels, like TRPM8 (Bautista et al., 2007; Colburn et al., 2007), which may allow for retained baseline behavioral sensitivity to cold in the absence of TRPA1. In heterologous expression systems or sensory neurons, murine TRPA1 is activated by noxious cold stimulation (Story et al., 2003; Karashima et al., 2009). However, it appears that TRPA1 is not necessary for baseline cold sensation behaviorally (Kwan et al., 2009; Knowlton et al., 2010). Naïve global TRPA1 knock-out mice were shown to have normal behavioral responses to cold, from -10^o^C to 20^o^C, and to radiant heat (Bautista et al., 2006). Interestingly, TRPA1 may still function in an uninjured state as a cold sensor; for example, TRPA1 is important in mediating the vascular response to environmental cold stimuli (Aubdool et al., 2014).

Although significant evidence has shown that TRPA1 contributes to hyperalgesia, these findings have not resolved whether the TRPA1’s contribution to somatosensation is tissue specific. It has been widely assumed that the effects of TRPA1 are mediated primarily by its presence in sensory neurons (Story et al., 2003; Corey et al., 2004; Julius, 2013); however, there are reports identifying TRPA1 expression in other cell types, including astrocytes and keratinocytes (Atoyan et al., 2009; Kwan et al., 2009; Shigetomi et al., 2012). Indeed, most studies of TRPA1 have used global knock-out mice (Kwan et al., 2006; Brierley et al., 2009; Kwan and Corey, 2009) or pharmacologic antagonists (Petrus et al., 2007; Eid et al., 2008; Kerstein et al., 2009), which lack cell-type specificity.

Given the possibility of TRPA1 expression in other cell types, we sought to isolate the contribution of TRPA1 expressed by primary sensory neurons to cutaneous sensation. To do so, we used *Advillin-Cre* mice (da Silva et al., 2011; Zurborg et al., 2011) to express Cre recombinase in sensory neurons. Advillin, an actin-binding protein involved in development of peripheral ganglion neurons, is a highly selective marker of peripheral sensory neurons within the dorsal and trigeminal ganglia (Hasegawa et al., 2007). Further, this occurs without apparent preference for particular subpopulations of sensory neurons (Zurborg et al., 2011). As such, this *Advillin-Cre* transgene is an effective method of driving Cre-mediated recombination in a majority (≥80%) of sensory neurons of all subclasses (Pagadala et al., 2013; Ranade et al., 2014). Crossing *Advillin-Cre* mice with a mouse containing loxP sites flanking the exons encoding the pore region of Trpa1 (*Trpa1*^fl/fl^ mice) allowed generation of sensory neuron-specific TRPA1 knock-out mice. Our findings show that sensory neuron-specific knock-out of TRPA1 significantly impairs mechanosensation, while leaving thermal sensation intact.

## Materials and Methods

### Animal generation and use

In order to address the role of *Trpa1* specifically within sensory neurons, we sought to create a conditional knock-out animal. Mice containing loxP sites flanking exons 22 through 24 of the *Trpa1* gene were used. Mice expressing two copies of this conditional knock-out allele are termed *Trpa1*^fl/fl^ mice (Zappia et al., 2016). Two independently-generated lines of *Advillin-Cre* mice have been described previously (da Silva et al., 2011; Zurborg et al., 2011). Heppenstall *Advillin-Cre* mice, described previously (Zurborg et al., 2011), contain a five copies of an inserted bacterial artificial chromosome (BAC) transgene containing Cre recombinase within the Advillin gene locus and its presumed regulatory elements. The second *Advillin-Cre* line, Wang *Advillin-Cre* mice, were described previously (da Silva et al., 2011) and were generated by targeted knock-in of the Cre allele into the second exon of the *Advillin* gene.

Both lines of the *Advillin-Cre* mice were crossed with the *Trpa1*^fl/fl^ mice (Zappia et al., 2016) to generate conditional knock-out animals; these *Advillin-Cre*, *Trpa1*^fl/fl^ mice are referred to here as *Adv^Cre^Trpa1*^fl/fl^ mice. Where applicable, *Adv^Cre^Trpa1*^fl/fl^ mice used throughout this manuscript are delineated by the line of *Advillin-Cre* from which they were generated (Hepp *Adv^Cre^Trpa1*^fl/fl^ and Wang *Adv^Cre^Trpa1*^fl/fl^). Importantly, expression of Cre alone in Advillin-expressing sensory neurons has been previously shown to have no effects on baseline sensory behaviors (Zurborg et al., 2011; Minett et al., 2012). As such, *Advillin-Cre*-expressing, *Trpa1*^+/+^ mice were used as controls for the conditional knock-out, *Adv^Cre^Trpa1*^fl/fl^ mice. Control mice from both Hepp and Wang colonies were used; the data were combined as no phenotypic differences were noted. Mating pairs in all cases consisted of a Cre^+^ male crossed with a Cre^-^ female, ensuring *Cre* transmission only from the male progenitor. Tissues from the appropriate animals were obtained and visualized for cells expressing functional Cre recombinase using an Ai9 fluorescent reporter mouse (*tdTomato*^LSL^), in which *tdTomato* is only expressed when a loxP-flanked STOP cassette is excised. Lastly, we used global *Trpa1* knock-out mice lacking TRPA1 in all tissues (Kwan et al., 2006) as a control.

In all instances, mouse genotype was confirmed by PCR. All methods and procedures were reviewed and approved by the Institutional Animal Care and Use Committee of the Medical College of Wisconsin. Animals were provided food and water *ad libitum* and housed on a 14/10 h light/dark cycle. Further, animal care adhered to the NIH Guide for the Care and Use of Laboratory Animals.

### Animal behavior

Behavioral tests of mechanical and thermal sensitivity were performed on the glabrous surface of the hindpaw of male and female mice at least eight weeks of age. No differences were noted between sexes, so these data were combined for all datasets. Experimenters were blinded to mouse genotype for all assays. Animals were acclimated to handling by the experimenter and were habituated to each applicable testing apparatus for an hour prior to test day (with one exception, as noted). Additionally, animals were habituated to the testing environment and apparatus for at least an hour on the day of testing.

For assays of mechanical sensitivity, mice were placed in small plexiglass enclosures on top of a wire mesh to allow probing of the plantar hind paw. Mechanical paw withdrawal thresholds were determined from responses to calibrated von Frey filaments (North Coast Medical), calculated using the Up-Down method as described previously (Dixon, 1980; Chaplan et al., 1994). Also, a 3.31 mN von Frey filament was applied 10 times to the plantar hindpaw, and the frequency of a withdrawal response was determined. Similarly, percent responses to both gentle punctate (0.7 mN von Frey filament) and dynamic (puffed cotton swab) stimuli were recorded; each stimulus was applied to both hindpaws 10 times, giving at least 30 s between stimuli. As a test of responsiveness to a noxious stimulus, a 25-gauge spinal needle was applied to the plantar hindpaw 10 times; each response was categorized into one of the following classifications: normal (withdrawal; a brief response), flutter, hold, lick, or no response. Again, the minimum interval between subsequent applications was 1 min, to avoid inducing sensitization. For all behavioral tests of mechanical sensitivity, left and right hindpaw responses were averaged for each animal.

To test heat sensitivity, mice were placed in small plexiglass enclosures on top of a glass plate, and a focal radiant heat source applied to the plantar hindpaw. The response latency to withdraw from the heat stimulus was quantified (Hargreaves et al., 1988). Cold sensitivity was measured using a two-temperature preference assay, consisting of two equally-sized, metal temperature-controlled metal floor plates (AHP1200-HCP, TECA Corp) enclosed within a 13” × 13” testing chamber. In the 5-min baseline period, animals were placed in the center of the chamber and allowed to explore freely, in order to test baseline activity and intrinsic chamber preference while both floor plates were held at room temperature (23^o^C). During this baseline phase, mice of both genotypes spent equivalent amounts of time on each of the testing chambers when both floor plates were held at 23^o^C. For the 5-min testing phase, the two floor plates were set at 20^o^C and 10^o^C, and mice were allowed to explore freely. The amount of time spent on each of the two plates was recorded and compared between genotypes.

### Sensory neuron isolation and culture

Sensory neurons were isolated from bilateral lumbar 1-6 dorsal root ganglia (DRGs) and cultured as follows. Male mice were anesthetized with isoflurane (Midwest Veterinary Supply) and euthanized via decapitation. Lumbar DRGs 1-6 were dissected bilaterally and placed in HBSS (Gibco, ThermoFischer Scientific). After the dissection, the isolated DRGs were transferred to a 50:50 mixture of DMEM and Ham’s F12 medium (Gibco). DRGs were next incubated at 37^o^C and 5% CO_2_ with 1 mg/mL collagenase type IV (Sigma) for 40 min, followed by a 45-min incubation with 0.05% trypsin (Sigma). Following chemical dissociation, the ganglia were washed and resuspended in a complete medium consisting of a 50:50 mixture of DMEM and Ham’s F12 medium supplemented with 10% heat-inactivated horse serum (Thermo Fisher Scientific), 2 mM L-glutamine (Thermo Fisher), 0.5% glucose (Sigma), and 0.2 Units Penicillin-Streptomycin (Thermo Fisher). The medium contained no added exogenous growth factors. Ganglia were manually triturated using a P1000 and P200 pipettor, and isolated neurons were plated onto glass coverslips coated with laminin (Sigma).

### Calcium imaging

Calcium imaging was performed in the presence of a synthetic extracellular normal HEPES (ENH) buffer containing: 150 mM NaCl, 10 mM HEPES, 8 mM glucose, 5.6 mM KCl, 2 mM CaCl_2_, and 1 mM MgCl_2_ (all from Sigma). After 18-28 h in culture, isolated cells were incubated for 45 min in 2% BSA and 2.5-μl/mL Fura-2 AM (Invitrogen), a dual-wavelength fluorescent calcium indicator, and washed for 30 min in ENH. Coverslips were then superfused with buffer at 6 mL/min and fluorescence images captured using a cCMOS camera (Zyla; Andor Technology LTD) using NIS Elements (Nikon Instruments) software. Fluorescence images were captured at both 340 nm (Ca^2+^ bound Fura-2) and 380 nm (unbound Fura-2). A ≥20% increase in 340 to 380 nm ratio from baseline measures was considered a response to a given stimulus, indicating an increase in intracellular calcium.

To assess functionality of TRPA1 expressed in sensory neurons, we used cinnamaldehyde (CINN; Sigma) as a TRPA1 agonist (Jordt et al., 2004), as it is more selective than allyl isothiocyanate, which can also activate TRPV1 (Everaerts et al., 2011). Following baseline measurements, coverslips were superfused with either the TRPA1-specific agonist, CINN (100 μM) for 3 min; a TRPV1 agonist, capsaicin (100 nM; CAP; Fluka, Sigma; Caterina, 2000) for 1 min; or both CINN followed by a 2-min wash, and then CAP. Following these stimuli, a 50 mM KCl (K+) solution was superfused to determine neuronal viability. Since the majority of TRPA1-expressing neurons are small-diameter neurons when observed via immunohistochemistry (Story et al., 2003), *in situ* hybridization (Kobayashi et al., 2005), or by using functional responsiveness in calcium imaging (Barabas et al., 2012), small-diameter neurons were the focus of this study. Imaging was not restricted to cells expressing Cre recombinase; all viable, small-diameter neurons within the imaging frame were used. Neurons were considered small if the cell body diameter was less than 27 μm; in mouse, this population typically corresponds to C fiber type somata (Dirajlal et al., 2003).

### Fluorescence imaging

Sensory neurons were isolated from lumbar DRGs and cultured as above; DRGs were obtained from *Adv^Cre^tdTomato*^LSL^ mice. The proportion of cultured DRG neurons expressing fluorescent tdTomato was calculated for *Adv^Cre^tdTomato* mice from both the Wang and Heppenstall colonies. Independently, DRGs from *Advillin-Cre* mice crossed with a reporter mouse expressing EGFP-tagged Cas9 were also isolated, sectioned, and visualized.

### Gene expression studies

Lumbar DRGs (1-6 bilaterally) were collected, and mRNA was isolated using TRIzol and the PureLink RNA Micro kit (Life Technologies). mRNA was reverse transcribed using the Superscript VILO master mix (Thermo Fisher Scientific), which includes both random hexamers and oligo dT for cDNA synthesis initiation. Real-time PCR was performed on a Mastercycler ep Realplex^2^ thermal cycler (Eppendorf) using TaqMan primers and probes (Life Technologies) for *Trpa1* and *Gapdh*. The pore region of *Trpa1* was amplified and detected using two TaqMan assays: Mm01227447_m1 to detect exons 23-24 and Mm00625259_g1 for exons 22-23.

### Data analysis

Paw withdrawal thresholds were compared between two groups using nonparametric Mann–Whitney *U* tests and among three groups using a Kruskal–Wallis test. All other behavioral assays were analyzed using parametric *t* tests for two groups, or a one-way ANOVA for three groups, with Sidak *post hoc* analysis. In calcium imaging experiments, the proportion of neurons responding to a given stimulus was compared between genotypes using a Fisher’s exact test, and the average magnitude of responses was compared using a Student’s *t* test. Gene expression studies were analyzed using one-way ANOVA with Sidak *post hoc*. Summarized data are reported as mean ± SEM. All data were analyzed using Prism 6 software (GraphPad), with an α of 0.05 set *a priori*. All statistical tests used, and resultant confidence intervals, are presented in Table 1.

**Table 1:** Statistical tests used within this manuscript

	Data structure	Type of test	Comparison	95% confidence interval
a	Non-normally distributed	Mann–Whitney *U* test		0.8792 to 2.533
b	Non-normally distributed	Mann–Whitney *U* test		-1.552 to 1.983
c	Normally distributed	*t* test		-32.19 to -12.11
d	Normally distributed	*t* test		-21.40 to 11.40
e	Normally distributed	*t* test		-21.01 to -6.262
f	Normally distributed	*t* test		-21.01 to -6.262
g	Normally distributed	*t* test		-23.05 to 7.592
h	Normally distributed	*t* test		-37.06 to 11.70
i	Normally distributed	*t* test		-6.135 to 5.468
j	Normally distributed	Two-way ANOVA		
		Sidak's multiple comparison test	Normal: WT vs *Adv^Cre^TRPA1*^fl/fl^	2.706 to 15.60
		Sidak's multiple comparison test	Hold: WT vs *Adv^Cre^TRPA1*^fl/fl^	-11.95 to 0.9492
		Sidak's multiple comparison test	Flutter: WT vs *Adv^Cre^TRPA1*^fl/fl^	-10.18 to 2.723
		Sidak's multiple comparison test	Lick: WT vs *Adv^Cre^TRPA1*^fl/fl^	-6.211 to 6.687
		Sidak's multiple comparison test	None: WT vs *Adv^Cre^TRPA1*^fl/fl^	-6.616 to 6.283
k	Normally distributed	*t* test		-0.4365 to 12.23
l	Normally distributed	*t* test		-12.79 to 11.03
m	Normally distributed	*t* test		-0.4637 to 1.700
n	Normally distributed	*t* test		-2.740 to 1.084
o	Normally distributed	One -way ANOVA		
	Normally distributed	Sidak's multiple comparison test	Exons 22-23: Wang control vs Wang *Adv^Cre^TRPA1*^fl/fl^	0.3935 to 1.390
	Normally distributed	Sidak's multiple comparison test	Exons 22-23: Hepp control vs Hepp *Adv^Cre^TRPA1*^fl/fl^	0.1303 to 1.188
	Normally distributed	Sidak's multiple comparison test	Exons 22-23: Hepp *Adv^Cre^TRPA1*^fl/fl^ vs Wang *Adv^Cre^TRPA1*^fl/fl^	-0.3397 to 0.7177
p	Normally distributed	One-way ANOVA		
	Normally distributed	Sidak's multiple comparison test	Exons 23-24: Wang control vs Wang *Adv^Cre^TRPA1*^fl/fl^	0.2601 to 1.200
	Normally distributed	Sidak's multiple comparison test	Exons 23-24: Hepp control vs Hepp *Adv^Cre^TRPA1*^fl/fl^	0.1235 to 1.063
	Normally distributed	Sidak's multiple comparison test	Exons 23-24: Hepp *Adv^Cre^TRPA1*^fl/fl^ vs Wang *Adv^Cre^TRPA1*^fl/fl^	-0.3632 to 0.5765
q	Normally distributed	χ^2^ w/Yates correction	Odds ratio, confidence interval reported	1.092 to 1.671
r	Normally distributed	Fisher's exact test	Odds ratio, confidence interval reported	5.460 to 11.06
s	Normally distributed	*t* test		-29.71 to 54.57
t	Normally distributed	Fisher's exact test	Odds ratio, confidence interval reported	0.5011 to 1.378
u	Normally distributed	*t* test		-57.15 to 126.7
v	Normally distributed	Fisher's exact test	Odds ratio, confidence interval reported	0.5210 to 0.9708
w	Normally distributed	*t* test		-37.03 to 69.37
x	Non-normally distributed	Kruskal–Wallis test	Dunn's *post hoc* multiple comparison test does not generate confidence intervals	
y	Normally distributed	One -way ANOVA		
		Sidak's multiple comparison test	WT vs *Adv^Cre^TRPA1*^fl/fl^	0.5382 to 20.57
		Sidak's multiple comparison test	WT vs TRPA1 KO	17.76 to 37.80
		Sidak's multiple comparison test	*Adv^Cre^TRPA1*^fl/fl^ vs TRPA1 KO	8.262 to 26.18

## Results

### Selective deficiency of TRPA1 within sensory neurons impairs baseline mechanical behavioral sensitivity

**Figure 1.**
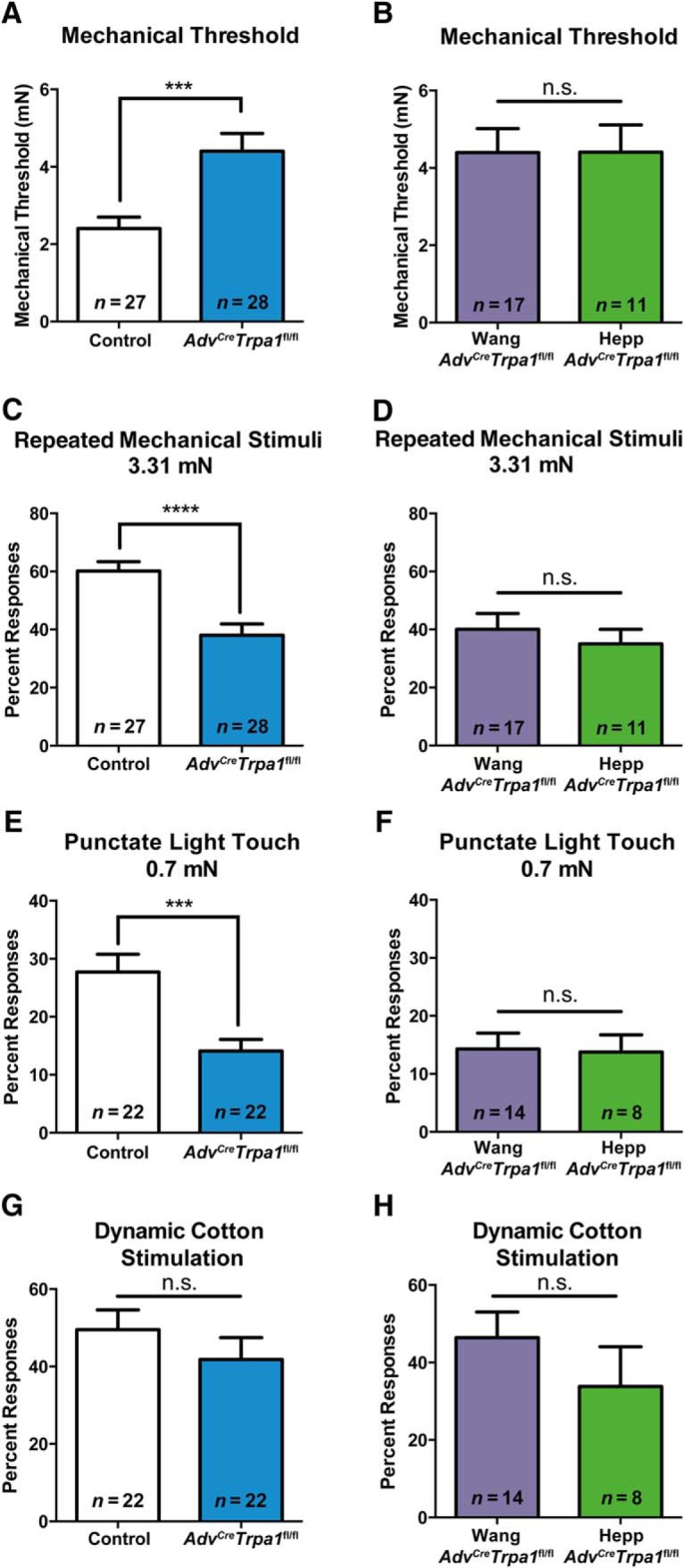
Deletion of TRPA1 from sensory neurons impairs mechanosensory behaviors. ***A***, In *Adv^Cre^Trpa1*^fl/fl^ animals, paw withdrawal thresholds were significantly elevated compared to controls. In the first panel, *Adv^Cre^Trpa1*^fl/fl^ animals are a combination of animals from both *Advillin-Cre* lines. ***B***, Paw withdrawal thresholds in *Adv^Cre^Trpa1*^fl/fl^ animals from the Wang and Heppenstall *Advillin-Cre* lines were similar. ***C***, Behavioral mechanical sensitivity, measured by repeated stimulation of the glabrous hindpaw with a 3.31 mN stimulus, was also impaired in the *Adv^Cre^Trpa1*^fl/fl^ animals. ***D***, Mechanical sensitivity to a repeated 3.31 mN stimulus was equivalent between *Adv^Cre^Trpa1*^fl/fl^ animals derived from each of the *Advillin-Cre* lines. ***E***, Sensitivity to a light touch (0.7 mN) stimulus was impaired in *Adv^Cre^Trpa1*^fl/fl^ mice, and this impairment was evident in both Wang and Heppenstall *Advillin-Cre* lines (***F***). ***G***, ***H***, Behavioral responsiveness to a dynamic light touch stimulus was not impacted by loss of sensory neuron TRPA1. The number of animals per group is denoted within each panel (***A***--***H***) of the figure. **p < 0.01; ***p < 0.001, n.s. denotes a nonsignificant comparison. Male and females were combined for all datasets because no differences were noted between the sexes.

In a test of mechanical sensitivity, *Adv^Cre^Trpa1*^fl/fl^ (combined from both Wang and Hepp Cre lines) mice displayed a nearly two-fold increase in paw withdrawal thresholds compared to controls (Fig. 1*A* ^a^). Importantly, similar impairments in mechanosensory phenotype were observed in both the Wang and Heppenstall *Advillin-Cre* lines (Fig. 1*B* ^b^). Similarly, there were no differences observed between controls (*Adv^Cre^Trpa1*^+/+^) from the Wang and Hepp lines and therefore, they were combined for all data sets. *Adv^Cre^Trpa1*^fl/fl^ mice also displayed reduced response frequencies when probed repeatedly with a 3.31 mN filament; again, there was a clear phenotypic overlap between the two *Advillin-Cre* lines, as there were no differences between *Adv^Cre^Trpa1*^fl/fl^ from the Wang and Hepp lines (Fig. 1*C* ^c^,*D*^d^). Additionally, there were no clear sex differences in any of these behavioral measures, so these data include combined outcomes from both sexes.

Responses to punctate light touch were reduced by 49% in *Adv^Cre^Trpa1*^fl/fl^ mice (Fig. 1*E* ^e^,*F*^f^). In contrast, responses to a dynamic light touch stimulus (cotton swab test) were not different between *Adv^Cre^Trpa1*^fl/fl^ and control mice (Fig. 1*G* ^g^,*H*^h^).

**Figure 2.**
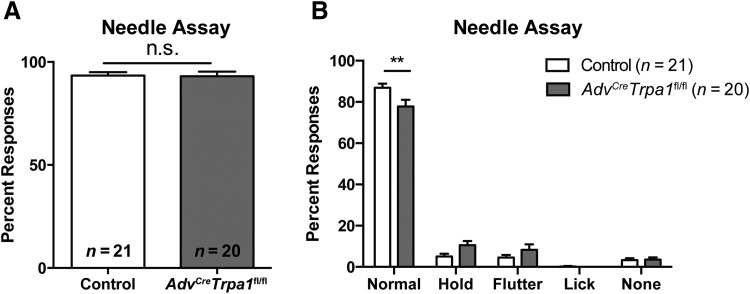
*Adv^Cre^Trpa1*^fl/fl^ mice exhibit altered responsiveness to a noxious stimulus. ***A***, Both control and *Adv^Cre^Trpa1*^fl/fl^ mice responded to a needle stimulus 93% of the time when all response types were included. ***B***, When responses to the needle stimulus were further categorized, *Adv^Cre^Trpa1*^fl/fl^ animals exhibited a decrease in the percentage of normal (brief withdrawal) responses. Mice in this figure are all from the Wang *Advillin-Cre* line. The number of mice per genotype is denoted within the figure. **p < 0.01.

We next tested for sensitivity to a noxious mechanical stimulus using a needle test. Interestingly, following application of a spinal needle to the plantar paw, we observed no deficit in *Adv^Cre^Trpa1*^fl/fl^ mice compared to controls (Fig. 2*A* ^i^). However, upon closer inspection, we observed that *Adv^Cre^Trpa1*^fl/fl^ mice exhibited a significant decrease in the percentage of normal responses to the stimulus and instead tended to have an increased propensity for holding their paw in the air or briefly fluttering the paw (Fig. 2*B* ^j^). Responses such as licking, holding, and fluttering of the hindpaw in response to a stimulus have previously been described as hyperalgesic-type responses (Hogan et al., 2004). However, anecdotally the *Adv^Cre^Trpa1*^fl/fl^ mice that held their paw up in this assay actually appeared uninterested, and these responses were unlike those of mice with paw inflammation, where it appears that the injured paw is being withheld and protected from further probing by the experimenter for example, by placing their paw out of reach on the cage wall, and often appearing simultaneously agitated. However, *Adv^Cre^Trpa1*^fl/fl^ mice rarely displayed these more exaggerated forms of holding their paw away from the experimenter.

### Sensory neuron-specific knock-out of TRPA1 does not impair behavioral responses to cold or heat

**Figure 3.**
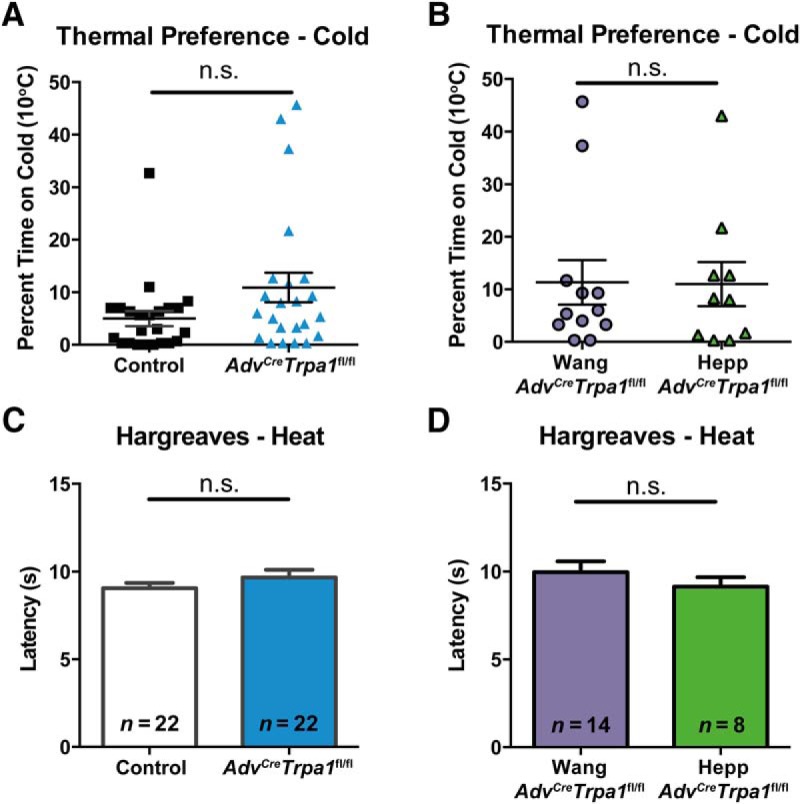
Thermosensory behaviors were not impacted by knock-out of TRPA1 from sensory neurons in naïve mice. ***A***, ***B***, As measured in a thermal preference assay, percent time spent on the colder of two plates (10^o^C or 20^o^C) was similar between control (*n* = 22) and *Adv^Cre^Trpa1*^fl/fl^ animals (*n* = 22 total; *n* = 14 Wang, *n* = 8 Hepp). ***C***, ***D***, Latency to paw withdrawal from a heat stimulus was not impaired in *Adv^Cre^Trpa1*^fl/fl^ mice. n.s. denotes no significant difference.

To test for impairment in cold sensitivity, we used a two-temperature preference assay, in which the floor of the testing chamber was divided in half into a mildly cool 20^o^C portion and a more noxious 10^o^C cold plate. Importantly, in this assay, behavioral cold aversion was unaltered in the *Adv^Cre^Trpa1*^fl/fl^ mice compared to controls, as *Adv^Cre^Trpa1*^fl/fl^ and controls spent an equivalent amount of time on the 10^o^C cold plate (Fig. 3*A* ^k^,*B*^l^). Interestingly, three of the *Adv^Cre^Trpa1*^fl/fl^ animals and one control mouse spent noticeably more time on the cold plate compared to the majority of the animals, implying interanimal variability independent of genotype. Despite this variability, it was clear that *Adv^Cre^Trpa1*^fl/fl^ mice retained the capacity to sense cold stimulation. Locomotor activity and exploratory drive was not impaired in the *Adv^Cre^Trpa1*^fl/fl^ mice, as these mice crossed between the two plates at the chamber midline a similar number of times during the baseline phase of cold testing (data not shown).

We next tested whether the *Adv^Cre^Trpa1*^fl/fl^ mice displayed any deficit in behavioral responses to thermal stimuli. Response latencies to a heat stimulus were not different between *Adv^Cre^Trpa1*^fl/fl^ and control mice (Fig. 3*C* ^m^). *Adv^Cre^Trpa1*^fl/fl^ mice from the Wang and Heppenstall Cre lines had similar heat response latencies, and both mouse lines were similar to control mice (Fig. 3*D* ^n^).

### Confirmation of Cre expression and deletion of Trpa1 in sensory neurons in Advillin-Cre+ mice

**Figure 4.**
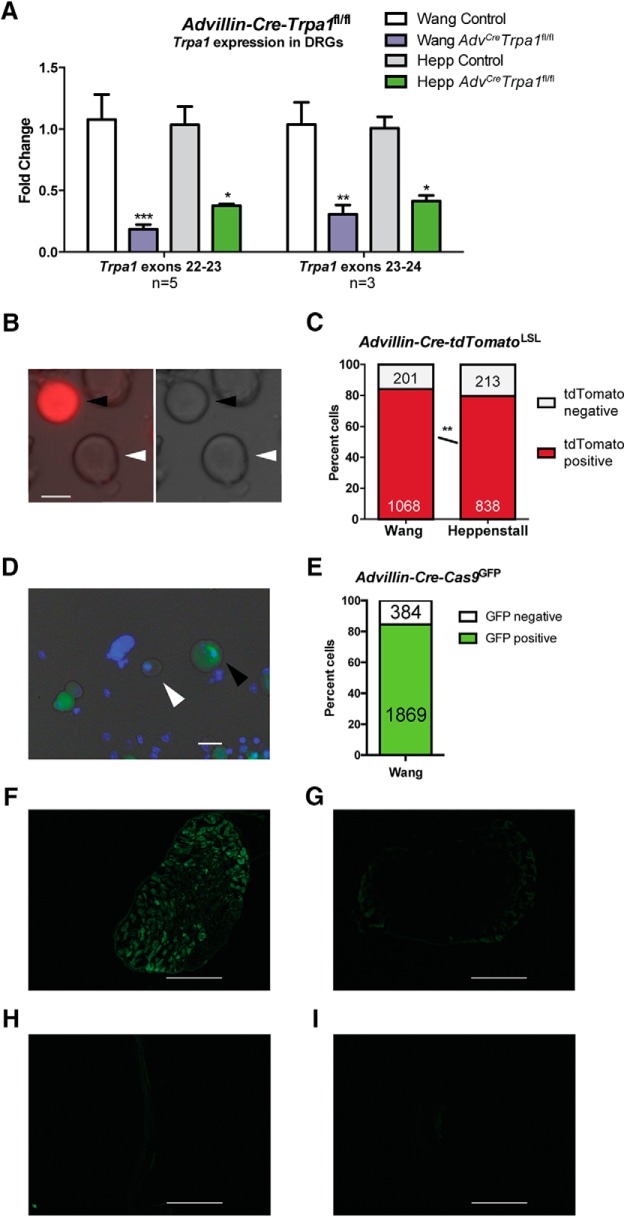
*Advillin-Cre* drivers induce functional Cre recombinase expression in sensory neurons. ***A***, RT-PCR of DRGs isolated from control and *Adv^Cre^Trpa1*^fl/fl^ animals showed a pronounced reduction in *Trpa1* mRNA in *Adv^Cre^Trpa1*^fl/fl^ animals. Two primer sets for *Trpa1* were used, each targeting the pore region of TRPA1. There was no significant difference comparing the *Adv^Cre^Trpa1*^fl/fl^ mice from the Wang and Hepp lines. Each bar represents results from three to five animals, each sample run in duplicate (*n* = 3 animals per genotype for exons 22-23, and *n* = 5 animals per genotype for exons 23-24). ***B***, Example of a Cre-negative, tdTomato-negative neuron (white arrowhead), and a Cre-positive, tdTomato-positive neuron (black arrowhead) from cultured *Adv^Cre^Trpa1*^fl/fl^ DRGs. On left is overlay of brightfield and fluorescent image. Scale bar, 20 μm. ***C***, Sensory neurons isolated and cultured from Wang *Advillin-Cre* reporter mice showed a slight but significant increase in the proportion of reporter-positive neurons compared to the Heppenstall *Advillin-Cre* line. Numbers represent the total number of tdTomato+ and tdTomato- neurons counted. ***D***, Example Cre+ (black arrowhead) and Cre- (white arrowhead) from *Advillin-Cre-Cas9^GFP^* mice, expressing an EGFP-tagged protein (Cas9) in the presence of Cre. Scale bar, 10 μm. ***E***, A total of 84.4% of sensory neurons from Wang *Advillin-Cre-Cas9^GFP^* DRGs were GFP-positive. ***F***, Fluorescence image of sectioned lumbar DRG isolated from an *Advillin-Cre-Cas9* animal. Scale bar, 50 μm. *G*, Fluorescent image of sectioned lumbar DRG from Cre-negative mouse. Scale bar, 50 μm. ***H***, ***I***, Fluorescent images of sectioned glabrous hindpaw skin from *Advillin-Cre-Cas9^GFP^* (***H***) and *Cre-negative-Cas9^GFP^* (***I***) mice. Scale bar, 50 μm. *p < 0.05; **p < 0.01; ***p < 0.001, n.s. denotes a nonsignificant comparison.

To confirm that Cre recombinase was functional in DRGs, we quantified the amount of *Trpa1* mRNA isolated from whole DRGs from control and *Adv^Cre^Trpa1*^fl/fl^ mice from each of the Cre lines. *Trpa1* transcript was significantly decreased by the conditional knock-out in both mouse lines (Fig. 4*A* ^o,p^). Here, there was a trend toward increased reduction in *Trpa1* transcript in the Wang Cre line than in the Heppenstall line, although no significant difference was detected. Results were similar between two PCR primer-probe sets, both targeting the pore-encoding region of the gene. This finding suggests deletion of *Trpa1* within sensory neurons of the *Adv^Cre^Trpa1*^fl/fl^ mice.

We concurrently isolated and cultured neurons from *Adv^Cre^tdTomato* reporter mice in order to visualize the proportion of cells expressing functional Cre. In the *Advillin-Cre* DRGs, approximately 80% of cultured neurons (all diameters included) were positive for tdTomato fluorescence (Fig. 4*B*,C ^q^), indicative of Cre activity in those cells. In animals from the Wang *Advillin-Cre* line, 84.2% of neurons were fluorescent, while only 79.8% of neurons from the Heppenstall *Advillin-Cre* line were fluorescent. Another reporter line (*Cas9-GFP*) was used to visualize Cre^+^ neurons in intact DRG. We first confirmed that 84.4% of isolated neurons from this reporter line (Wang) expressed GFP, indicating active Cre recombinase (Fig. 4*D*,*E*). Next, we show a similar proportion of Cre^+^ neurons in sectioned *Advillin-Cre-Cas9^GFP^* DRGs (Fig. 4*F*). Importantly, this Cre activity was not observed in *Cre^-^* DRG sections (Fig. 4*G*), nor was Cre activity present in either *Advillin-Cre^+^* or *Cre^-^* skin sections (Fig. 4*H*,*I*) or spinal cord sections (data not shown).

### Functional loss of TRPA1 within sensory neurons

**Figure 5.**
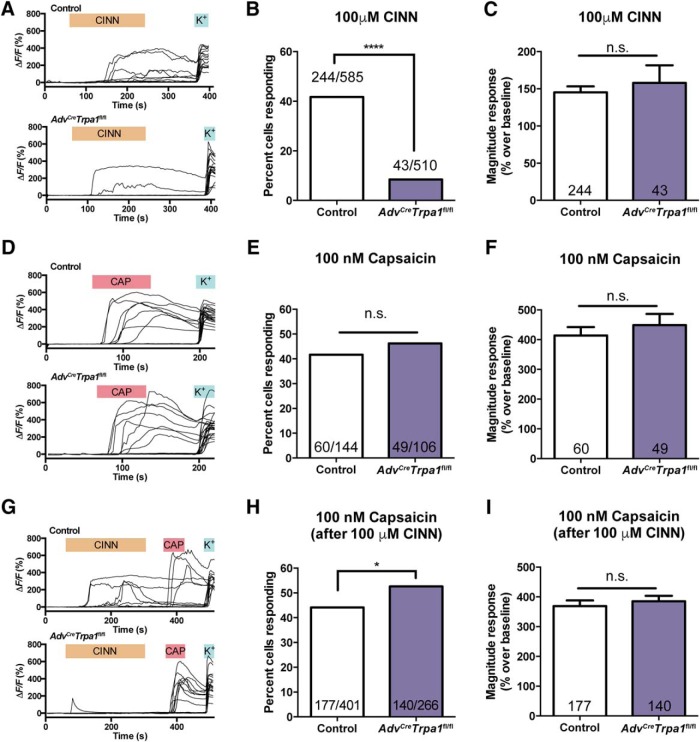
Calcium imaging of small-diameter control and *Adv^Cre^Trpa1*^fl/fl^ sensory neurons indicates significant TRPA1-mediated functional deficits. Sensory neurons tested via calcium imaging were all from the Wang *Advillin-Cre* line. ***A***, Example traces of increasing intracellular calcium in several individual neurons following application of 100 μM cinnamaldehyde (CINN). *ΔF/F* (%) indicates the percent increase of Fura-2 340/380 nm fluorescence normalized to baseline for each individual cell. K+ indicates 50 mM KCL used to depolarize neurons. ***B***, The proportion of sensory neurons responding to 100 μM CINN is significantly decreased in *Adv^Cre^Trpa1*^fl/fl^ mice. *N* listed on the figure indicate the number of cells responding to the stimulus over the number of cells tested. This experiment was replicated twice for each genotype (two animals per genotype); results were combined. ***C***, Among neurons respond to 100 μM CINN, cells from control and *Adv^Cre^Trpa1*^fl/fl^ mice had a similar magnitude calcium response. ***D***, Sample traces of Fura-2 fluorescence in control and *Adv^Cre^Trpa1*^fl/fl^ small-diameter neurons after stimulation with 100 nM capsaicin (CAP). ***E***, ***F***, When stimulated with 100 nM capsaicin, neurons from control and *Adv^Cre^Trpa1*^fl/fl^ mice responded with a similar frequency (***E***) and magnitude of response; two animals per genotype (***F***). ***G***, Example traces of *ΔF/F* (%) (percent increase in Fura-2 fluorescence over baseline) in control and *Adv^Cre^Trpa1*^fl/fl^ exposed sequentially to both 100 μM CINN and 100 nM capsaicin. ***H***, Following administration of 100 μM CINN, sensory neurons from *Adv^Cre^Trpa1*^fl/fl^ mice exhibited a slightly although significantly increased proportion of responsiveness (two animals per genotype). ***I***, Even after 100 μM CINN exposure, sensory neurons from control and *Adv^Cre^Trpa1*^fl/fl^ mice responded with equivalent magnitude of calcium responses to a 100 nM capsaicin stimulus. *p < 0.05; ****p < 0.0001, and n.s. denotes a nonsignificant comparison.

To more directly address the functional effect of the conditional knock-out on sensory neurons, we used calcium imaging to assess the responsiveness of isolated sensory neurons to the TRPA1 agonist, cinnamaldehyde (CINN). Given the broad phenotypic overlap between the Wang and Heppenstall lines, and because there was increased tdTomato reporter expression in the Wang *Advillin-Cre* line, imaging was only performed on sensory neurons from the Wang *Adv^Cre^Trpa1*^fl/fl^ animals. Small-diameter sensory neurons isolated from Wang *Adv^Cre^Trpa1*^fl/fl^ animals were much less likely to respond (≥20% over baseline) to 100 μM CINN compared to control neurons (Fig. 5*A*,*B*^r^). Importantly, the conditional knock-out line displayed a five-fold reduction in the percentage of neurons responding to CINN compared to control neurons, corresponding to the expression of Cre in 80% of sensory neurons (Fig. 4*B*). In the 8% of neurons that remained responsive, the magnitude of calcium influx remained unchanged compared to control responses (Fig. 5*C* ^s^). Analysis of CINN responsiveness was limited to small-diameter neurons but was not restricted by expression of TRPV1. In a separate experiment, there was no impairment in responsiveness to the TRPV1 agonist, capsaicin, as 42-46% of small neurons from either cohort responded (Fig. 5*D*,*E* ^t^,*F*^u^). Some coverslips were treated first with CINN and then capsaicin; interestingly, among cells preexposed to CINN administration, there was a slightly increased prevalence of neurons responding to capsaicin in the *Adv^Cre^Trpa1*^fl/fl^ mice (Fig. 5*G*,*H* ^v^,*I*^w^).

### Conditional TRPA1 knock-out mice display incomplete disruption of mechanosensory ability

**Figure 6.**
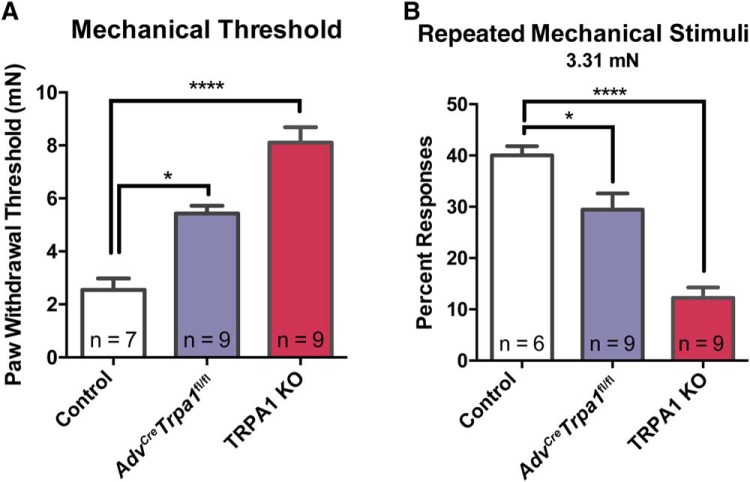
*Advillin-Cre*-mediated knock-out of TRPA1 from sensory neurons results in incomplete impairment of mechanosensation. ***A***, Paw withdrawal thresholds from both *Adv^Cre^Trpa1*^fl/fl^ and global TRPA1 KO animals were elevated compared to controls. Thresholds in *Adv^Cre^Trpa1*^fl/fl^ mice were 113% higher than those of control mice; global KO mice displayed a 218% increase over control mice. ***B***, Similar, withdrawal frequency to a repeated stimulus was significantly impaired in both control and global KO mice. Mice in this particular experiment were habituated to the testing apparatus an hour before testing, but did not receive exposure to the apparatus prior to that day. The number of mice tested per genotype is denoted within the figure. *p < 0.05; ****p < 0.0001.

Given the incomplete elimination of TRPA1 from the sensory neurons of the *Adv^Cre^Trpa1*^fl/fl^ animals (approximately 80%, both at the level of mRNA and functionally via calcium imaging), we next compared the behavioral mechanosensory deficit of the *Adv^Cre^Trpa1*^fl/fl^ to that of global TRPA1 knock-out mice (Kwan et al., 2006). Given the restriction of TRPA1 deletion from only sensory neurons, we would not anticipate sensory phenotypes beyond those identified in global TRPA1 knock-outs. Here, we observed that global TRPA1 knock-out mice displayed a greater deficit in mechanosensory behaviors than the *Adv^Cre^Trpa1*^fl/fl^ mice (Fig. 6 ^x,y^), although both *Adv^Cre^Trpa1*^fl/fl^ and global TRPA1 mice displayed elevated paw withdrawal thresholds (Fig. 6*A*) and decreased responsiveness to a 3.31 mN stimulus (Fig. 6*B*) compared to controls.

## Discussion

These data demonstrate that sensory neuron-specific knock-out of TRPA1 induces clear mechanosensory deficits, an observation which is in strong concordance with previous findings in global TRPA1 knock-out mice (Kwan et al., 2006), while aversion to noxious cold using a behavioral preference assay remained intact. These results distinctly implicate sensory-neuron TRPA1 in baseline mechanical sensitivity *in vivo*. Further studies utilizing this mouse line will be valuable in addressing other sensory neuron-specific functions of TRPA1.

Our findings of only partial mechanosensory impairment compared to global knock-out mice are in line with the incomplete ablation of TRPA1 from all sensory neurons, as evidenced by both gene expression studies and functional assessment via calcium imaging. However, this is understandable given that Cre-mediated recombination under the Advillin promoter was only observed in 80% of cultured sensory neurons, and a corresponding 80% reduction in CINN-responsive neurons in the *Adv^Cre^Trpa1*^fl/fl^ animals. That is, the small amount of remaining TRPA1 within a subpopulation (20%) of sensory neurons may largely mediate this apparent incomplete deficit when compared to the global knock-outs. Similarly, others have shown an incomplete deficit in proprioception when ablating Piezo2 using an *Advillin-CreERT2* driver, while Cre drivers that were more specific to proprioceptive populations of neurons produced a more severe deficit (Woo et al., 2014). Nonetheless, these data demarcate a clear role for sensory neuron expression of TRPA1 in mechanosensation.

These findings also indicate that sensory neuron expression of TRPA1 is not required for behavioral cold sensation and subsequent aversion in an uninjured state. More specifically, an 80% reduction of TRPA1 from sensory neurons does not produce a deficit in noxious (10^o^C) cold sensation; this finding is consistent with previous reports in non-injury states, showing that TRPA1 is not required in the mediation of baseline cold detection (Bautista et al., 2006; Knowlton et al., 2010) or C and Aδ fiber activation by cold stimulation from 32^o^C to 2^o^C (Kwan et al., 2009). However, from these data, we cannot exclude that TRPA1 may still be activated by noxious or tissue-damaging cold in naïve animals and may thereby have an impact in other paradigms of cold sensitivity. For example, global TRPA1 knock-out mice show starkly impaired nocifensive jumping responses to a 0^o^C cold plate (Karashima et al., 2009). Therefore, it is possible that TRPA1 may signal a nociceptive response to intense cold that may injure tissue, but that ion channels other than TRPA1 are sufficient to signal cold detection and aversion during non-injury conditions.

Importantly, our findings served as a comparison between two independently-generated *Advillin-Cre* mouse lines. The complementary findings between two methods driving TRPA1 deletion provide strong evidence that this deletion is responsible for the sensory deficits. This is of particular importance due to the sometimes unnoticed, unintentional effects of Cre recombinase expression, or from deleterious impacts of either targeted recombination or random insertion during transgenic mouse generation. In this case, random insertion of *Cre* using a BAC transgene (Heppenstall line) or targeted insertion (Wang line) generated similar mechanosensory deficit phenotypes when crossed with the *Trpa1*^fl/fl^ mice.

Advillin has also been found to be expressed in Merkel cells of the glabrous epidermis and has been used as a marker of such within the skin (Ranade et al., 2014; Whiteley et al., 2015). It is therefore reasonable to be cautious when interpreting the effects of an *Advillin-Cre* mouse line, particularly when the gene of interest is expressed in other cell types. Importantly, an extensive search did not detect *Trpa1* mRNA transcripts in epidermis isolated from mouse skin, which would include both keratinocytes and Merkel cells (Zappia et al., 2016). Therefore, it is unlikely that the present results are due to deletion of TRPA1 in Merkel cells.

Further, we show that *Advillin-Cre* expression was not observed in keratinocytes of the skin, and such expression was also absent from spinal cord sections (data not shown). This important confirmation of tissue specificity of Cre expression suggests that any other potential sites of TRPA1 expression (e.g., keratinocytes, spinal cord astrocytes) would be unaffected in this particular study. As such, the deficit in mechanosensitivity in these *Advillin-Cre-Trpa1^fl/fl^* mice can be attributed to the deletion of *Trpa1* selectively from sensory neurons. That is, although the present experiments cannot exclusively preclude any nonneuronal expression of TRPA1, the results do in fact justify the many studies that have implicated neuronal TRPA1 in mechanosensation.

Given its abundant expression in sensory tissues, including sensory ganglia and inner ear hair cells (Story et al., 2003; Corey et al., 2004; Julius, 2013), it has been largely assumed that expression of TRPA1 within sensory neurons is responsible for the phenotypes observed in TRPA1-deficient animals. However, sensory neurons do not act in isolation. In addition to the clear role of primary sensory neurons in cutaneous sensation, keratinocytes have recently become recognized as potential players within mechanosensation, thermosensation and nociception (Peier et al., 2002; Chung et al., 2004; Denda et al., 2007; Baumbauer et al., 2015; Pang et al., 2015). Given their location, epidermal keratinocytes are uniquely poised to respond to external stimuli, and indeed are capable signaling this information to sensory neurons through release of several paracrine mediators, including ATP, prostaglandins, endothelin-1, interleukins, or other cytokines (Southall et al., 2003; Lumpkin and Caterina, 2007; Huang et al., 2008; Olaru and Jensen, 2010; Azorin et al., 2011; Shi et al., 2013; Zappia et al., 2016).

The expression of TRPA1 within nonneuronal tissues has been proposed and debated. For example, there are reports of TRPA1 expression in mouse and human skin (Atoyan et al., 2009; Kwan and Corey, 2009); however, in other studies, expression of *Trpa1* mRNA was not detected in mouse epidermis (Liu et al., 2013; Zappia et al., 2016). Thus, it has remained important to directly address whether the *in vivo* functional role of TRPA1 in mediating baseline mechanical sensitivity is mediated by sensory neurons or nonneuronal cell types. Importantly, our data present strong evidence that deletion of TRPA1 selectively from a majority (80%) of sensory neurons is sufficient to induce a strong mechanosensory deficit both behaviorally and at the level of isolated sensory neurons. These data also suggest that even if TRPA1 is expressed in other cell types, this extraneuronal TRPA1 is not sufficient to maintain mechanical sensory function. As such, TRPA1 within sensory neurons is of intrinsic importance in mediating mechanical sensation.

In all, these results provide evidence from two complementary and independent *Advillin-Cre* mouse lines indicating that TRPA1 specifically expressed within sensory neurons is essential for normal baseline sensitivity to multiple types of mechanical stimuli. These mouse lines should be a valuable asset for mechanistically dissecting the sensory neuron TRPA1 contribution to a variety of other chronic pain and itch conditions.

## Synthesis

Reviewing Editor: Mary Heinricher, Oregon Health & Science University Decisions are customarily a result of the Reviewing Editor and the peer reviewers coming together and discussing their recommendations until a consensus is reached. When revisions are invited, a fact-based synthesis statement explaining their decision and outlining what is needed to prepare a revision will be listed below. The following reviewer(s) agreed to reveal their identity: Alfredo Ribeiro-da-Silva, Stuart Bevan

It is well-accepted that TRPA1 participates in somatic sensation and pain. However, TRPA1 is not restricted to primary afferents, and the whether functions attributed to TRPA1 reflect that expressed in peripheral sensory neurons has not been established. In this manuscript, the authors used a Cre-lox approach to achieve sensory neuron-specific deletion of TRPA1, and demonstrate, using two Cre driver lines, that TRPA1 deletion in the majority (approx.. 80%) of dorsal root ganglion neurons impairs behavioral responses to mechanical stimuli, while responses to heat or cold are unaffected. Importantly, concordant results were obtained in the two transgenic lines, which were independently-generated.

Overall, the paper is clearly written and the behavioral studies appropriately rigorous. There are nonetheless aspects that could be improved.

1) It would be helpful to see some raw data in some of the figures where only summary results are shown. For example:

a) Representative traces of increases in intracellular calcium concentration in response to cinnamaldehyde and capsaicin. This should include responsive and unresponsive neurons.

b) Images of DRG from AdvCretdTomato reporter mice, showing positive and negative cells.

c) Quantify the cas9-GFP data and provide a fuller description of the results and the rationale for examining these mice. Also, Figure 4C could be better quality, and a scale bar should be provided in the image itself.

2) It is surprising that data on heat and cold sensitivities of TRPA1 global KO was not provided (or omission justified based on the literature, e.g., Bautista et al., 2006, Cell).

3) A rationale for the advillin-Cre driver lines should be provided in the Introduction. Also, a stronger argument (or supportive data) for specificity of the deletion to primary afferents is needed. It has been reported that TRPA1 is expressed in astrocytes (Shigetomi et al. 2011) and it is not impossible that TRPA1 is expressed in keratinocytes (see work from the Stucky group). Ideally, if tissue from these lines is available, it should be possible to show the lack of reporter in astrocytes/keratinocytes.

Minor comments:

It was very difficult to figure out the n's for the different experiments. These should be clearly outlined.

The interval between the noxious needle stimuli should be better defined. (What constitutes “sufficient time”?)

The statement that mice “appeared uninterested” in the needle stimulus is interesting. What was qualitatively different in their responses?

In the sensory neuron dissociation protocol, reword “DRGs were next the ganglia at 37...”

The wording “functional similarity to other cold sensors” is unclear. What is the intention here?
